# Efficient vitreolysis by combining plasmin and sulfur hexafluoride injection in a preclinical study in rabbit eyes

**Published:** 2012-09-19

**Authors:** Wei-Chi Wu, Chi-Hsien Liu, Chih-Chun Chen, Nan-Kai Wang, Kwan-Jen Chen, Tun-Lu Chen, Yih-Shiou Hwang, Lien-Min Li, Chi-Chun Lai

**Affiliations:** 1 Department of Ophthalmology, Chang Gung Memorial Hospital, Taoyuan, Taiwan; 2 Chang Gung University, College of Medicine, Taoyuan, Taiwan; 3 Graduate Institute of Biochemical and Biomedical Engineering, Chang Gung University, Taoyuan, Taiwan; 4 Microscopy Core Laboratory, Chang Gung Memorial Hospital, Taoyuan, Taiwan

## Abstract

**Purpose:**

To investigate the efficacy of plasmin and sulfur hexafluoride (SF_6_) on the vitreoretinal junction, as well as the long-term safety in the eye and effect on the recipient’s general health after application in the eye.

**Methods:**

The study design included four groups of rabbits with three animals in each group. Group 1 received an intravitreal injection (IVI) of plasmin and SF_6_ in the right eye; group 2 received an IVI of plasmin in the right eye; group 3 received an IVI of SF_6_ in the right eye; and group 4 received an IVI of balanced salt solution in the right eye, which served as a normal control. Long-term safety (up to approximately three months) after plasmin and/or SF_6_ injection was evaluated morphologically by clinical examination, histology, and immunohistochemistry, and functionally by electroretinograms (ERGs). General health evaluations after intravitreal injection included the assessment of weight gain, food intake, body temperature, and complete blood count analysis.

**Results:**

Plasmin plus SF_6_ injection resulted in complete posterior vitreous detachment (PVD), whereas plasmin or SF_6_ injection alone resulted in only partial PVD. Balanced salt solution did not induce PVD. Eighty days after intravitreal injection, there were no major differences among the eyes of the three groups of animals compared with the normal control animals upon clinical evaluation, or regarding retinal morphology and ERGs. The lenses examined remained clear for up to 80 days following the intravitreal injection of plasmin plus SF_6_, except one eye in the plasmin-treated group. ERGs decreased transiently one week after intravitreal injection in groups 1 through 3, but animals recovered fully to normal status afterward. General health was not affected after the injection of plasmin plus SF_6_.

**Conclusions:**

Efficient vitreoretinal separation could be achieved, and an acceptable long-term safety profile was noted after plasmin plus SF_6_ injection in the eye. No major ocular toxicity or systemic toxicity was found after the injection of plasmin plus SF_6_. These results provide good support for the future clinical use of plasmin plus SF_6_ for treatment of a variety of vitreoretinopathies.

## Introduction

Vitreous traction on the retina can be a significant pathological factor in certain retinopathies, including central retinal vein occlusion, pediatric vitreoretinopathy, diabetic retinopathy, age-related macular degeneration, and cystoid macular edema [1-7]. Studies have shown that patients with posterior vitreous detachment (PVD), which is characterized by a lack of vitreous traction on the retina, had a better visual prognosis in certain retinopathies such as retinal vessel occlusion and age-related macular degeneration [4,7]. Relief of vitreous traction by the induction of PVD is theoretically helpful for these retinopathies.

Plasmin is a serine protease that mediates the fibrinolytic process and modulates the extracellular matrix [8]. It hydrolyzes a variety of glycoproteins, including laminin and fibronectin, both of which are present at the vitreoretinal interface and are thought to play a key role in vitreoretinal attachment [9,10]. Plasmin enzyme has been proven to cause vitreous liquefaction and PVD [11-17]. Pharmacological vitreolysis with microplasmin, a truncated form of plasmin, increases vitreous diffusion coefficients [18] and oxygen levels in the vitreous [19]. Therefore, plasmin might be useful in treating a variety of retinopathies because it reduces vitreous traction and retinal ischemia.

In cases without cellular attachment in the vitreoretinal junction, plasmin injection could weaken vitreoretinal adhesion and result in PVD. However, in cases with tough vitreoretinal adhesion with a cellular component, plasmin injection alone results in partial PVD [20]. This condition is termed “anomalous PVD,” and is associated with a worse outcome. Therefore, the plasmin enzyme was used clinically, mainly as an adjuvant, to reduce vitreoretinal adhesion during vitrectomy surgery [11-15,21-23]. With the application of a plasmin enzyme, PVD was less traumatic than when mechanical methods alone were used [24]. To increase its efficiency in cleaving vitreoretinal adhesion, Sebag was the first to propose the concept of combination pharmacologic vitreolysis therapy [25]. Combination therapy could work more effectively than a single agent in conditions characterized by firm vitreoretinal adhesion.

Several animal studies have shown a good safety profile for plasmin when used in the eye [20,26-28]. Most of the studies used plasmin as a single agent to induce PVD and were conducted over short study periods. Very few studies have addressed the combination vitreolysis technique [20,29]. The long-term safety of plasmin combined with other agents in the eyes remains unknown. The purpose of this study was to investigate the long-term effect of plasmin plus sulfur hexafluoride (SF_6_) on vitreoretinal adhesion, ocular safety, and the recipient’s general health after application in the eye.

## Methods

### Evaluation

The effect of PVD produced by the plasmin enzyme was investigated by transmission and scanning electron microscopy (TEM and SEM, respectively). The long-term safety profile of an intravitreal plasmin injection was evaluated by examining morphological as well as functional changes in the retina. Clinical examinations included slit lamp examinations, indirect ophthalmoscope, and fundus photos. Morphological studies included retinal histology and immunohistochemistry (IHC) by various antibodies that recognized specific layers of retinal cells. Functional studies of the retina used electroretinograms (ERGs) to identify functional changes in the retina after plasmin injection. An evaluation of general health after intravitreal injection included the assessment of weight gain, food intake, body temperature, and complete blood count (CBC) analysis.

### Animals

Japanese white rabbits (1.5–1.7 kg) were used in this study. The animals were purchased from the Animal Health Research Institute, Council of Agriculture (Executive Yuan, Jhunan, Taiwan) and were housed in the animal care facilities of the Chang Gung Memorial Hospital, Taoyuan, Taiwan. Animal handling was performed in accordance with the regulations at Chang Gung Memorial Hospital for the use of experimental animals and the Association for Research in Vision and Ophthalmology statement for the use of animals in Ophthalmic and Vision Research. The execution of this project followed the guidelines and standards of Good Laboratory Practice.

### Grouping of the animals and intravitreal injection

The right eye of each rabbit in group 1 received a pars plana injection of 1 unit of human plasmin (0.1 ml reconstituted in sterile balanced salt solution [BSS]; CalBiochem, La Jolla, CA) plus 0.5 ml of SF_6_ in the mid vitreous cavity. The right eye of each rabbit in group 2 received an intravitreal injection of 1 unit of human plasmin (0.1 ml) only. The right eye of each rabbit in group 3 received an intravitreal injection of 0.5 ml of SF_6_ only. The left eye of each animal in these three groups received an injection of 0.1 ml BSS. Animals in group 4 received a BSS injection in the right eye and no injection in the left eye. Each group consisted of three animals. The rabbits were anesthetized with intramuscular injections of 1.5 mL/kg of an equal volumemixture of 2-(2.6-xylidino)-5.6-dihydro-4H-1.3-thiazine-hydrochloride, methylparaben (Rompun; Bayer AG, Leverkusen, Germany) and 50 mg/ml ketamine (Ketomin; Nang Kuang Pharmaceutical Co., Tainan, Taiwan). Topical anesthesia (Alcaine; Alcon-Couvreur, Puurs, Belgium) was administered to reduce the animals’ discomfort [30].

The intravitreal injection was performed 2 mm posterior to the limbus while the eye was being observed under a surgical microscope (M691; Wild Heerbrugg, Heerbrugg, Switzerland), with the help of a prism lens. Care was taken to avoid damage to the lens and the retina during the injection.

### Clinical observations and electrophysiological examination

Slit-lamp (SL-15; Kowa, Tokyo, Japan) examinations and indirect ophthalmoscopy (Omega 500; Heine, Herrsching, Germany) were performed. The degree of conjunctival congestion was evaluated by the Cornea and Contact Lens Research Unit (CCLRU) grading scale [31]. The severity of conjunctival redness ranged from very slight (grade 1) to slight (grade 2), moderate (grade 3), and severe (grade 4). External photos and color fundus photos were obtained to document the status of the cornea, conjunctiva, lens, vitreous, and retina after plasmin plus or without SF_6_ injection. For ERG recordings, the rabbits were anesthetized, their pupils were dilated, and a topical anesthetic was applied to the cornea. After 1 h of dark adaptation, ERGs were recorded with an ERG recording system (RETIport ERG; Roland Consult, Brandenburg, Germany) at baseline and at 1, 3, 7, 14, 28, and 80 days after the intravitreal injections. ERGs were recorded with a contact lens electrode that contained light-emitting diodes as a stimulator and that was connected to an electrode on the forehead. A ground electrode was attached to the ear. Amplitudes and implicit times of a- and b-waves were evaluated. These protocols have been published previously [30]. The luminance of the stimulus was 3 cd/m^2^, with a duration of 10 ms. Scotopic 0-dB ERGs were recorded with a standard white flash and a dark background. Twenty responses elicited by identical flashes applied at 10-s intervals were averaged in the dark-adapted state.

### Histological and electron microscopic examination

Eighty days after intravitreal injection, all animals were simultaneously sacrificed by an overdose of anesthetics. After enucleation, the eyes were opened with a razor blade, which was used to penetrate the vitreous adjacent to the pars plana to ensure rapid penetration of the fixative. Care was taken to avoid damage to the adjacent retina and lens. Morphological examinations by histology, SEM, and TEM were performed in each eye. One third of the retina was sectioned for IHC, one third for SEM examination, and one third for retinal histology and TEM examination. Therefore, each eye was used for all three morphological analyses.

For TEM, after fixation by a mixture of 3% glutaraldehyde and 2% paraformaldehyde, the tissue was dehydrated in an ethanol series, postfixed in 1% osmium tetroxide, and embedded in epoxy resin (Epok 812; Oken, Tokyo, Japan). Semithin sections were stained with 0.5% toluidine blue. Ultrathin sections were stained for contrast with 8% uranyl acetate and lead citrate, and were analyzed using electron microscopy (H7500; Hitachi, Tokyo, Japan). The observers were blinded to group classification when they interpreted the morphological data. SEM was performed to verify the TEM findings.

### Immunohistochemistry with confocal microscopy

IHC was used to visualize cells in different retinal layers 80 days after intravitreal injection. The integrity of the intermediate filament proteins of Müller cells and rods were verified. A protocol that was published in a previous study was used with modifications [30]. In brief, after the cornea, lens, and vitreous were removed, the eye cup was cut into three pieces, and one of these pieces was used for the IHC study. The retinas were fixed in 4% paraformaldehyde overnight. They were then incubated in 30% sucrose (USB Corp., Cleveland, OH) overnight at 4 °C, embedded in an optimal cutting temperature compound (Sakura Finetek, Torrance, CA), and sectioned with a microtome cryostat (CM3050S; Leica, Wetzlar, Germany). The sections were placed on slides that had been coated with silane (Muto PureChemicals, Tokyo, Japan) to promote adhesion of the sections to the glass surface. Samples were blocked with 1% BSA (in PBS) for 60 min after washing in PBS. After removal of the blocking serum, the following primary antibodies were added: antivimentin (1:1; Dako, Glostrup, Denmark) and antirhodopsin (1:50; Santa Cruz Biotechnology, Santa Cruz, CA). IgG-fluorescein isothiocyanate was used as a secondary antibody. The resulting sections were then viewed under confocal microscopy (TCSSP2; Leica, Wetzlar, Germany).

### Body temperature, food Intake, and weight gain

Body temperature, food intake, and weight gain were viewed as indicators of general health in the rabbits. Body temperature and food intake were measured on a daily basis, and the weight of each animal was measured at least every 2 weeks and on the day of anesthesia.

### Complete blood count

Before and after the experiment, a CBC was taken to detect signs of infection, anemia, or abnormalities in the blood. Blood from the ear was collected for analysis.

### Statistical evaluation

We compared ERG results between the study eyes and the control eyes before and after intravitreal injection and among the different treatment groups. The results among the different groups were compared using ANOVA and the Dunnett post hoc test. The Wilcoxon signed-rank test was used to compare ERG results at baseline and after treatment, as well as to compare results between study eyes and control eyes. Amplitudes and implicated times of the a- and b-waves were analyzed by group mean comparisons. CBC data were compared before and after intravitreal injection and among different treatment groups. p<0.05 was considered statistically significant.

## Results

### Clinical examinations and electroretinograms

**Figure 1:**
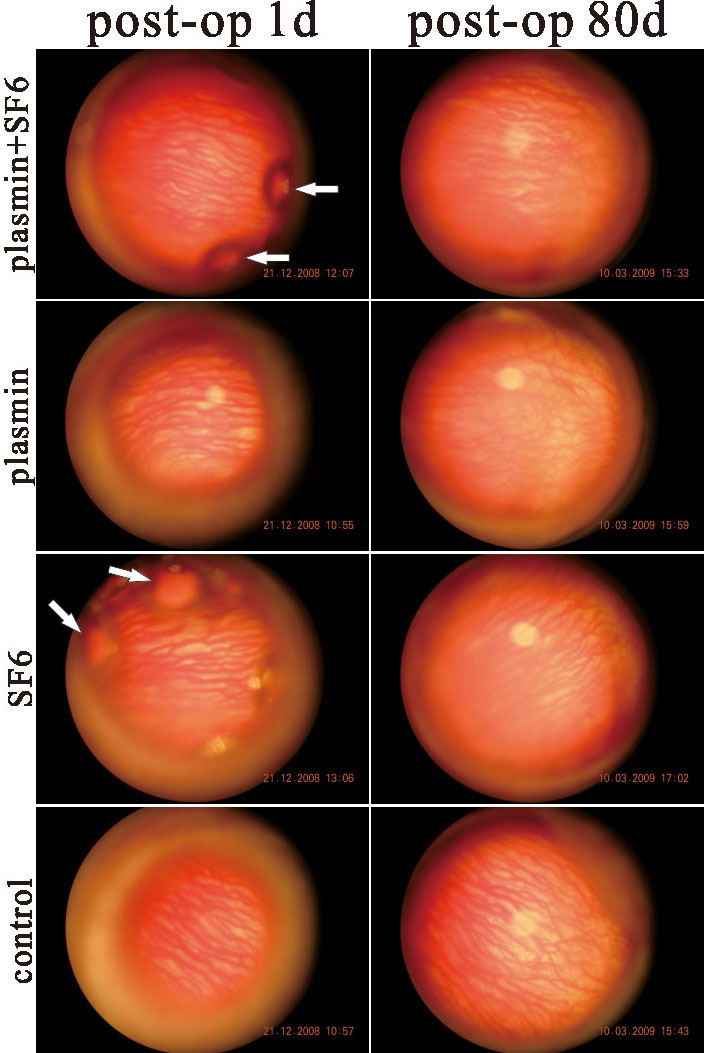
Fundus after intravitreal injection of plasmin and/or SF_6_. Up to 80 days following the intravitreal injection of plasmin and/or SF_6_, the dilated fundus revealed no signs of vitreous opacity, retinal detachment, vessel occlusion, or retinal necrosis. Arrows indicate the long-acting gas SF_6_ after intravitreal injection. Post-op 1d represents one day after intravitreal injection and post-op 80d represents 80 days after intravitreal injection.

**Figure 2:**
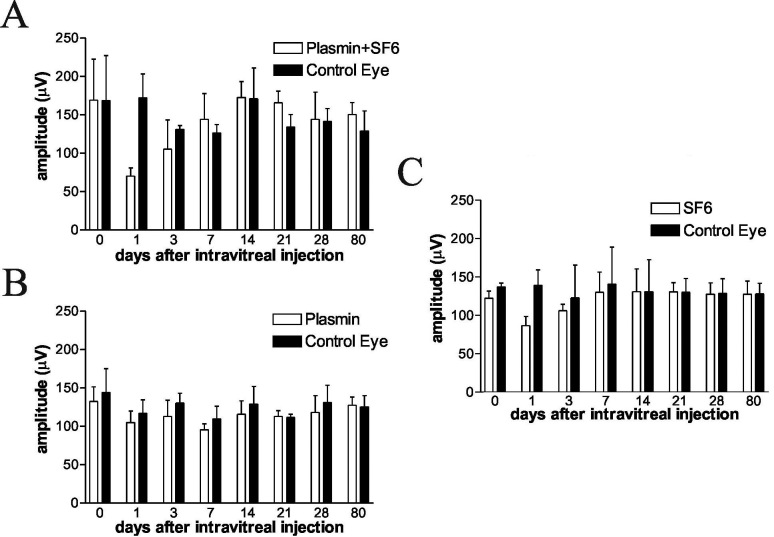
Electroretinogram b-wave changes after the injection of **A**: plasmin and SF_6_ (group 1), **B**: plasmin (group 2), or **C**: SF_6_ (group 3). The right eye of each rabbit in group 1 received a pars plana injection of 1 unit of human plasmin and 0.5 ml of SF_6_. The right eye of each rabbit in group 2 received an intravitreal injection of 1 unit of human plasmin (0.1 ml) only. The right eye of each rabbit in group 3 received an intravitreal injection of 0.5 ml of SF_6_ only. The left eye of each animal in these three groups received a 0.1 ml balanced salt solution (BSS) injection. Electroretinogram (ERG) data showed a transient decrease in the b-wave amplitude within one week after the injections in groups 1, 2, and 3. But the decrease did not reach statistical significance (p value=0.109, 0.285, 0.109 from group 1 to 3 [day 1]; p=0.285, 0.285, 0.593 from group 1 to 3 [day 3]; p=0.593, 0.285, 0.593 from group 1 to 3 [day 7]).Each group consisted of three animals. Data were expressed as mean±SD. One week after the injections, the amplitude returned to baseline.

At 7, 14, 28, and 80 days after intravitreal injection, animals were anesthetized, and external photographs were taken with a digital camera. The degree of conjunctival congestion was assessed by the CCLRU grading scale. Signs of wound infection and corneal epithelial defects were recorded. The degree of conjunctival congestion was similar between the experimental and control eyes. After surgeries, moderate to severe conjunctival congestion lasted for approximately two weeks, after which time the redness decreased gradually. There was no significant difference in the degree of conjunctival redness assessed by the CCLRU grading scale up to 80 days after surgery among these treatment groups. None of these eyes developed corneal or conjunctival infection by the end of the experiment. A mild cellular reaction was noted in the anterior chamber after intravitreal injection of plasmin plus or without SF_6_, but the reaction cleared in all eyes within one week. The lens remained clear up to 80 days following the intravitreal injection of plasmin plus SF_6_, except one eye in the plasmin-treated group. Dilated fundus examinations revealed no signs of vitreous opacity, retinal detachment, vessel occlusion, or retinal necrosis in any animal (Figure 1). ERG data showed a transient decrease in a- and b-wave amplitude within one week after the injections in groups 1, 2, and 3. At one week after the injections, the amplitude returned to baseline. Representative ERG b-wave changes are shown in Figure 2.

### Histology of the retina

**Figure 3:**
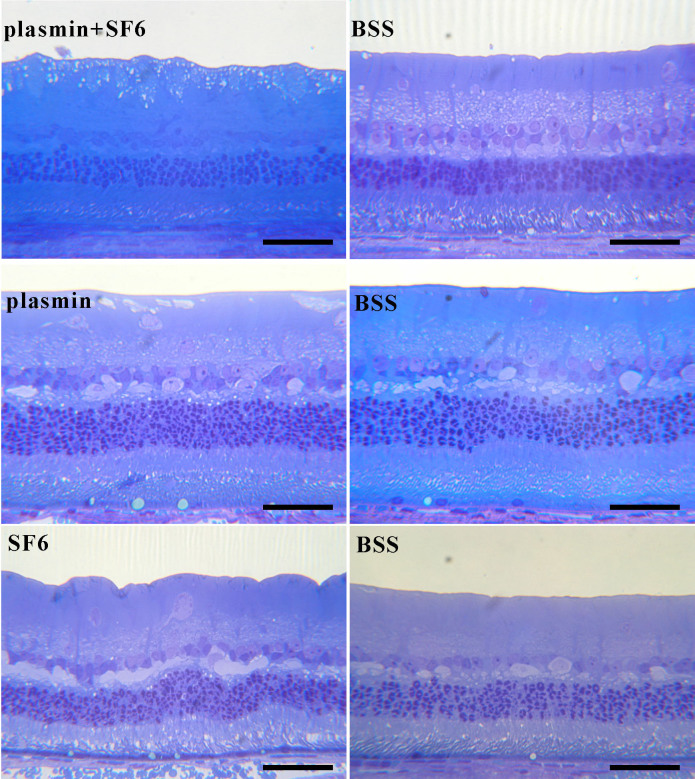
Histology of the retina among the three treatment groups 80 days after intravitreal injection. The retinal morphology of plasmin and/or SF_6_ treated eyes was similar to that of the balanced salt solution (BSS)-treated eyes (control eyes). The scale bar represents 50 μm.

There were no noticeable differences in the histology of the retina among the three treatment groups by the end of this study (Figure 3). The retinal morphology of plasmin plus or without SF_6_–treated eyes was similar to that of the control eyes. Some vacuole changes could be seen in all groups of animals, even in the normal control group. However, no significant morphological changes, including retinal structure deformation, thinning, or retinal layer loss, were seen in a specific group of animals. Therefore, such morphological changes may have been associated with the tissue handling or the normal physiologic apoptosis or aging changes of the retina.

**Figure 4:**
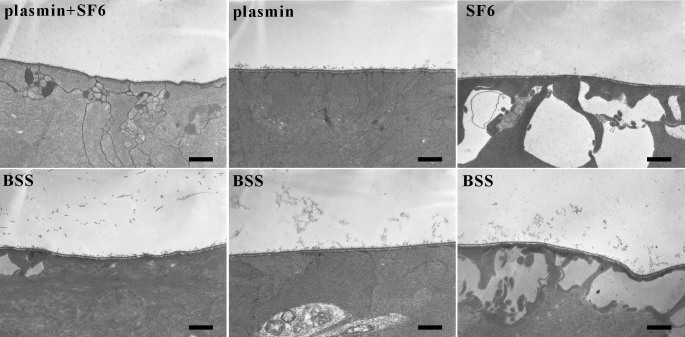
Transmission electronic microscope images of the eyes treated with plasmin and/or SF_6_. Dense collagen fibrils were still attached to the internal limiting membrane (ILM) in the balanced salt solution (BSS)-treated eyes (control eyes). In contrast, eyes treated with plasmin or SF_6_ showed a cleaner ILM with limited adhesion of collagen fibrils. Eyes treated with plasmin and SF_6_ were free from adhesion of collagen fibrils. The scale bar represents 1 μm.

TEM was used to investigate the presence of vitreoretinal adhesion and to characterize the ultramorphology in different retinal layers after plasmin plus or without SF_6_ treatment. Dense collagen fibrils were still attached to the internal limiting membrane (ILM) in the control eyes. In contrast, eyes treated with plasmin or SF_6_ alone showed a cleaner ILM with limited adhesion of collagen fibrils. Eyes treated with plasmin plus SF_6_ were free from adhesion of collagen fibrils (Figure 4). These results showed that BSS-treated eyes did not undergo PVD. Partial PVD was observed in the plasmin injection group (group 2) and the group receiving the long-acting gas, sulfur hexafluoride (group 3). However, the plasmin enzyme plus the long-acting SF_6_ gas (group 1) produced complete PVD. The ultrastructure in the retinal pigment epithelium, the outer segments of photoreceptors, the inner segments of photoreceptors, the mitochondria in the inner segments, and cells in the outer nuclear layer showed normal morphology in all four treated groups. SEM and TEM findings were consistent.

### Immunohistochemistry

**Figure 5:**
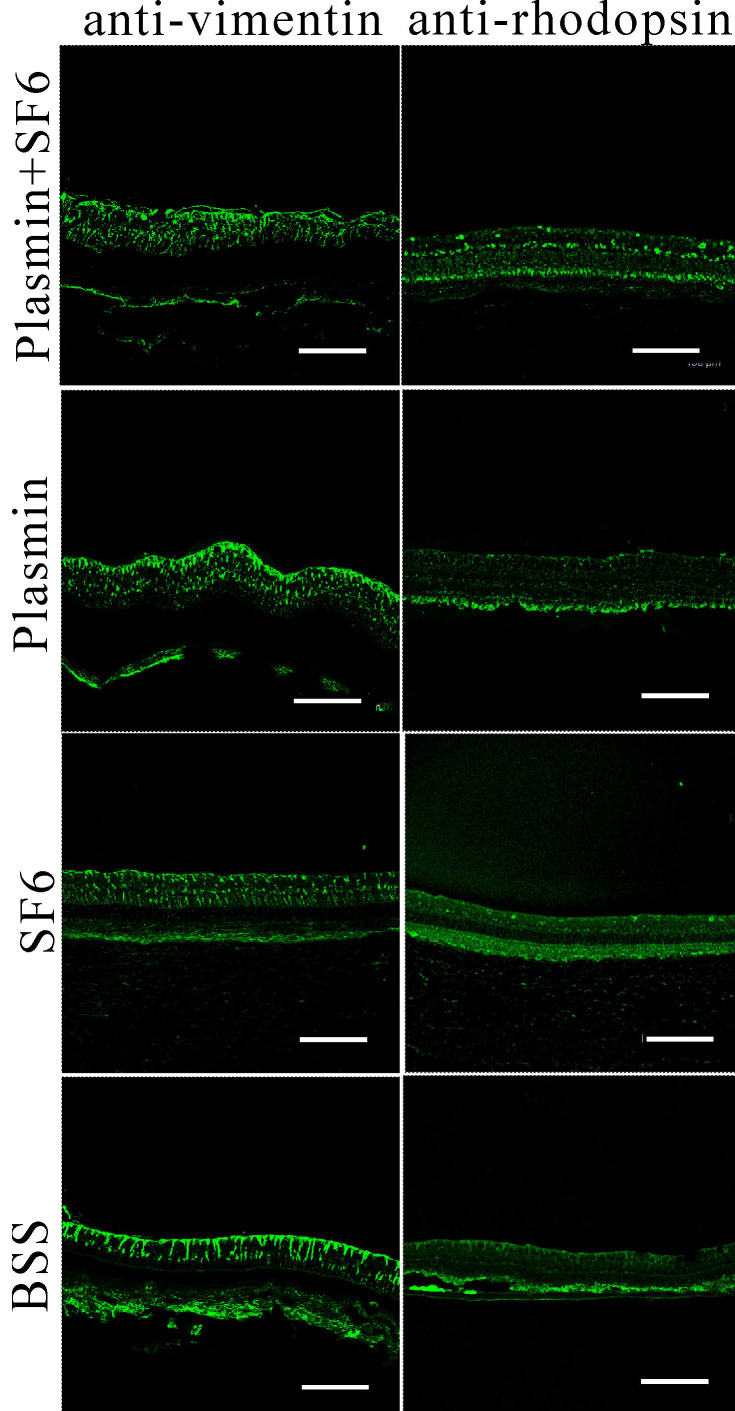
Representative images from immunohistochemistry 80 days after intravitreal injection of plasmin and SF_6_ (group 1), plasmin (group 2), SF_6_ (group 3), or balanced salt solution (control group). There were no significant differences in the retinal cell components between eyes receiving plasmin and/or SF_6_ and balanced salt solution (BSS). The scale bar represents 150 μm.

IHC using antibodies that recognized different layers of cells in the retina was performed to compare the difference between treated and control eyes. We did not find any major differences in the IHC results, indicating that the specific cells within the retina were not affected by plasmin plus or without SF_6_ treatment (Figure 5).

### Body temperature, food Intake, weight gain, and complete blood count

We did not find any differences in body temperature, food intake, or weight gain among rabbits receiving plasmin plus SF_6_, plasmin alone, or SF_6_ alone. There were no major differences in the CBC before and after intravitreal injection among the three groups of animals (data not shown).

## Discussion

It is important to assess the long-term safety of plasmin plus SF_6_, including ocular safety and systemic safety, before beginning the clinical application of these agents. Our data also showed good efficacy of PVD induction by plasmin plus SF_6_. Our clinical observations, as well as our morphological and functional studies on the eye, revealed good safety profiles of plasmin plus the long-acting gas SF_6_ when injected into the eye. The rabbits’ general health was not affected by the injection of plasmin plus SF_6_. This study has provided important preclinical information regarding the application of plasmin plus SF_6_ in treating a variety of retinopathies.

Plasmin plus other agents or surgery is necessary to produce a complete PVD in clinical situations with tight vitreoretinal adhesion. In the clinical trial of vitreomacular adhesion treated with intravitreal microplasmin (ThromboGenics Ltd., Dublin, Ireland), a truncated form of plasmin, PVD occurred 8% to 44% of the time, depending on the dose of microplasmin used [32]. Repeated injection of microplasmin (up to three times) increased the incidence of PVD to 58% [32]. These data suggest that even with the use of plasmin and after multiple enzyme injections, the induction of PVD did not reach 100%. However, the plasmin enzyme alone could not produce a complete PVD in some retinal disorders with firm vitreoretinal adhesion, such as diabetic retinopathy [20]. Plasmin injection alone in diabetic retinopathy weakens the vitreoretinal adhesion and most often results in partial PVD only, which is an even more dangerous situation with a worsened clinical outcome [33,34]. Additional procedures, such as vitrectomy or the combination of other agents, are needed to induce a complete PVD in retinopathy with prominent cellular proliferation and attachment of the vitreoretinal junction. SF_6_ injection alone has been shown to induce PVD clinically [35,36]. Combining plasmin with the long-acting gas SF_6_ might be more effective in cases with particularly strong vitreoretinal adhesion and may induce a complete vitreoretinal separation, unlike that achieved by the use of a single agent. In addition, this procedure is less aggressive than combining plasmin and vitrectomy.

Although the half-life of plasmin is short, its effect could last for long time. For instance, cataract formation might not be visible immediately after the injection of plasmin, but may appear later. Therefore, the long-term effect is important if we are going to use plasmin and SF_6_ to treat a variety of vitreoretinopathies with tougher vitreoretinal adhesion in the future. Hikichi et al. [29] was the first to use plasmin and SF_6_ to induce PVD in rabbit eyes. They found effective PVD induction after the use of plasmin combined with SF_6_. They observed a short-term effect of combined vitreolysis by plasmin and SF_6_ up to seven days; we extended the follow-up to almost three months. In addition to ocular safety evaluation, we added the evaluation of systemic safety. Our findings suggest the potential of combining plasmin and SF_6_ because of the desired clinical effect and good safety profile associated with such treatment.

In the current study, we observed transient ERG changes following the injection of plasmin. After one week, the ERG returned to normal baseline levels. We hypothesize that this phenomenon is associated with the pressure changes following intravitreal injection. The pressure changes might be related to the properties and the volume of the agents injected into the vitreous. We noted mild transient inflammation after the injection of plasmin into the rabbit eyes. The intraocular pressure was high immediately after intravitreal injection, but gradually returned to normal [37]. This indicates that the agents injected into the vitreous caused transient photoreceptor dysfunction, but the ERG values returned to pretreatment levels shortly after the injection. Eyes in group 4 received the lowest injected volume of all the groups and BSS did not cause an inflammatory reaction after its injection in the eyes. These two factors could contribute to the lack of ERG changes after intravitreal injection in these animals. Transient and mild inflammation could be encountered following the injection of plasmin plus or without SF_6_. Cataracts could also arise following intravitreal injection of plasmin, although the incidence is not high. This result could be attributed to the intravitreal injection or the effect of plasmin on the lens. The combination of plasmin plus SF_6_ seems to be well tolerated in the eye and was not associated with increased complications. Yet, our study is limited by the small number of animals used. No definite conclusions could be drawn, although the initial results are promising.

Notably, the PVD effect produced by plasmin is dose and time dependent [38]. In the clinical trial of vitreomacular traction syndrome treated with microplasmin, increased exposure and doses augmented the incidence of PVD induction [39]. Therefore, sufficient doses of plasmin and time for action are required to produce the complete effect of vitreoretinal separation. Insufficient doses or time for action will produce either no separation at all or only partial vitreoretinal separation, a condition that is associated with poor prognoses, as mentioned above.

In conclusion, our results suggest an efficient vitreoretinal separation and a good long-term safety profile for plasmin plus SF_6_ injection into the eye. No major systemic toxicity was found after the injection of plasmin plus SF_6_. These results provide support for the future clinical use of plasmin plus SF_6_ to treat a variety of vitreoretinopathies with tougher vitreoretinal adhesion.
